# Supplementary material for: Splice-Junction-Based Mapping of Alternative Isoforms in the Human Proteome
Source: Cell Rep. Author manuscript; Available in PMC 2020 Jan 15. (PMC6961840; doi:10.1016/j.celrep.2019.11.026)
Supplement: 2 [file NIHMS1546469-supplement-2.zip › DF1/PXD010154/Testis/Testis_48_PIAS3_LGGDFLSSLPLHEYPPAFPLGADIQGLDLFSFLQTESQVMVMSFR.pdf]

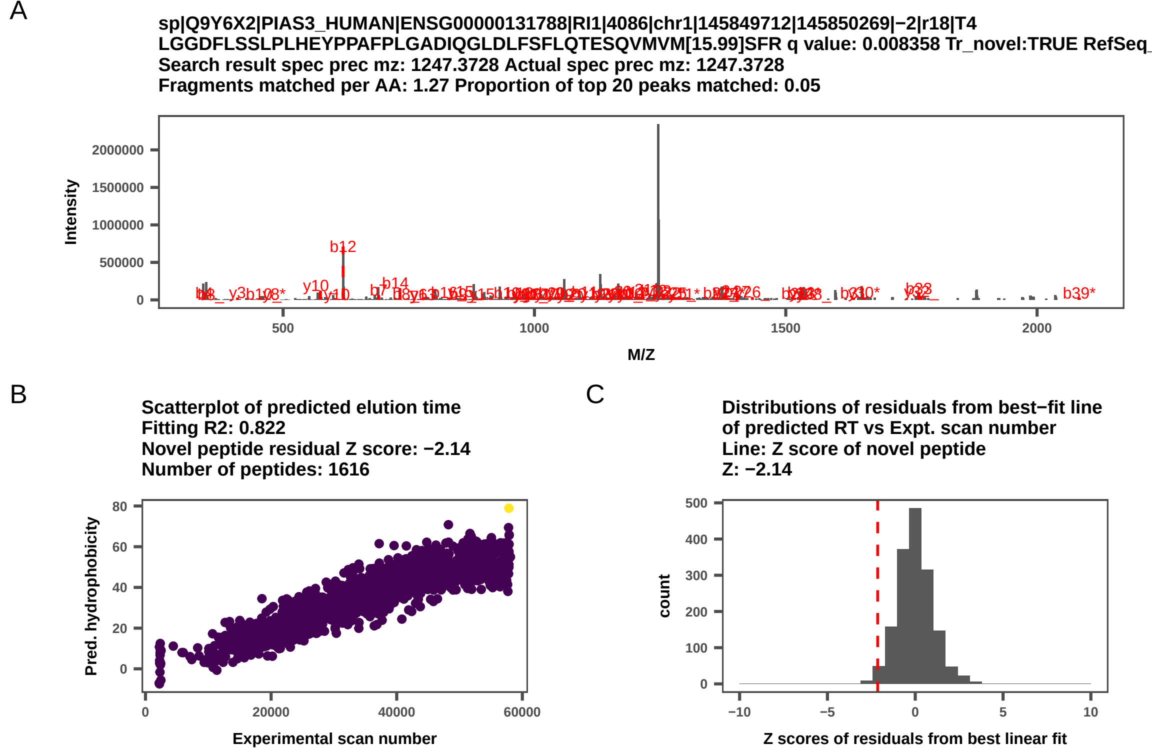
A
sp|Q9Y6X2|PIAS3_HUMAN|ENSG00000131788|RI1|4086|chr1|145849712|145850269|−2|r18|T4
LGGDFLSSLPLHEYPPAFPLGADIQGLDLFSFLQTESQVMVM[15.99]SFR q value: 0.008358 Tr_novel:TRUE RefSeq_
Search result spec prec mz: 1247.3728 Actual spec prec mz: 1247.3728
Fragments matched per AA: 1.27 Proportion of top 20 peaks matched: 0.05
Intensity
2000000
1500000
1000000
500000
0
b12
b14
y10
b39*
500
1000
1500
2000
M/Z
B
Scatterplot of predicted elution time
Fitting R2: 0.822
Novel peptide residual Z score: −2.14
Number of peptides: 1616
Pred. hydrophobicity
80
60
40
20
0
0
20000
40000
60000
Experimental scan number
C
Distributions of residuals from best−fit line
of predicted RT vs Expt. scan number
Line: Z score of novel peptide
Z: −2.14
count
500
400
300
200
100
0
−10
−5
0
5
10
Z scores of residuals from best linear fit
